# Archival data review of intimate partner homicide-suicide in Jamaica, 2007 – 2017: focus on mental health and community response

**DOI:** 10.26633/RPSP.2019.99

**Published:** 2019-11-27

**Authors:** Audrey M Pottinger, Althea Bailey, Nickiesha Passard

**Affiliations:** 1 Department of Child and Adolescent Health The University of the West Indies Mona, Kingston Jamaica Department of Child and Adolescent Health, The University of the West Indies, Mona, Kingston, Jamaica.; 2 Department of Community and Psychiatry The University of the West Indies Mona, Kingston Jamaica Department of Community and Psychiatry, The University of the West Indies, Mona, Kingston, Jamaica.

**Keywords:** Intimate partner violence, mental health, Jamaica, Violencia de pareja, salud mental, Jamaica, Violência por parceiro íntimo, saúde mental, Jamaica

## Abstract

**Objective.:**

To identify social and behavioral patterns and mental health concerns associated with intimate partner homicide-suicide (IPHS) in Jamaica through an analysis of media and police reports.

**Methods.:**

This was an archival data review of police records and print, radio, and television reports of IPHS incidents from January 2007 – June 2017 in Jamaica. The 27 cases found were qualitatively analyzed using pre-identified codes and open coding to generate themes and patterns.

**Results.:**

A prevalence rate of 0.1 per 100 000 was determined. In all cases, males were the homicide-offender. Sociodemographic patterns associated with IPHS incidents—age, personality traits, choice of weapon, and time of occurrence—were consistent with previous findings. Common triggers were offender obsession, sexual jealousy, and fear of separation. Despite reports of mental health concerns in both male and female partners, neither the couples nor community members sought help prior to the homicide-suicide.

**Conclusion.:**

These findings demand a change in cultural attitudes toward domestic disputes and mental health concerns, and a redefining of the community’s responsibility in IPHS. The warning signs associated with IPHS should be part of existing violence and suicide prevention programs.

## RESUMEN

**Objetivo.:**

Identificar los patrones sociales y de comportamiento y los problemas de salud mental asociados con el homicidio de la pareja íntima seguido de suicidio (HPIS) en Jamaica mediante el análisis de los informes de medios de comunicación y policiales.

**Métodos.:**

Revisión de datos de los archivos policiales y de los informes publicados en la prensa escrita, la radio o la televisión sobre HPIS en Jamaica entre enero de 2007 y junio de 2017. Los 27 casos identificados se analizaron cualitativamente mediante códigos preidentificados y códigos abiertos adicionales a fin de generar temas y patrones.

**Resultados.:**

Se determinó una tasa de prevalencia de 0,1 por 100 000 habitantes. En todos los casos, los autores del homicidio fueron hombres. Los patrones sociodemográficos asociados con los incidentes –edad, rasgos de personalidad, arma empleada y momento del suceso– coincidieron con los encontrados en incidentes anteriores. Fueron desencadenantes frecuentes la conducta obsesiva del agresor respecto de su pareja, los celos sexuales y el miedo a la separación. A pesar de la presencia de informes sobre problemas de salud mental tanto en los hombres como en las mujeres, ni las propias parejas ni los miembros de la comunidad buscaron ayuda antes del homicidio seguido de suicidio.

**Conclusión.:**

Estos hallazgos exigen cambiar las actitudes culturales hacia las disputas domésticas y los problemas de salud mental, y redefinir la responsabilidad de la comunidad ante estos incidentes. Las señales de advertencia asociadas con el HPIS deben ser parte de los programas de prevención de la violencia y el suicidio.

## RESUMO

**Objetivo.:**

Identificar os padrões sociais e comportamentais e as questões de saúde mental associados aos homicídios por parceiro íntimo seguidos por suicídio (HPIS) na Jamaica, a partir de análise da mídia e de inquéritos policiais.

**Métodos.:**

Revisão de inquéritos policiais e de relatos publicados na imprensa escrita, no rádio ou na televisão sobre HPIS na Jamaica de janeiro de 2007 a junho de 2017. Os 27 casos identificados foram analisados qualitativamente utilizando códigos pré-identificados e codificação aberta para gerar temas e padrões.

**Resultados.:**

Foi detectada uma taxa de prevalência de 0,1 por 100 000 habitantes. Em todos os casos, os autores do homicídio eram homens. Os padrões sociodemográficos associados aos incidentes de HPIS – idade, traços de personalidade, arma utilizada e momento do evento – coincidiram com os encontrados em estudos anteriores. Obsessão por parte do agressor, ciúme sexual e medo da separação foram gatilhos frequentes. Apesar dos relatos de problemas de saúde mental em ambos os parceiros, tanto do sexo feminino quanto do masculino, nem o casal nem os membros da comunidade procuraram ajuda antes do homicídio seguido do suicídio.

**Conclusão.:**

Estes resultados indicam a necessidade de mudanças culturais na atitude frente a disputas domésticas e problemas de saúde mental e de uma redefinição da responsabilidade da comunidade quanto a HPIS. Os sinais de alerta associados a HPIS devem ser contemplados em programas de prevenção da violência e do suicídio.

Though incidents of intimate partner homicide-suicides (IPHS) are rare (< 0.001% of any population), they are singularly devastating to families and communities (1, 2). IPHS is a challenge facing most health systems. Research examining its magnitude has found rates ranging from 0.01 – 1.7 per 100 000 (3, 4) and suggests it may be higher in countries with high homicide rates (3). Most published data on the topic are epidemiological, highlighting risk factor identification (3). Few studies examine the role of mental disorders and intimate partner violence (IPV; 5). In the Caribbean, despite much media and public interest in IPHS, relevant research is scant (6).

The World Health Organization (WHO) has recognized the growing body of knowledge on the magnitude, patterns, and risk factors associated with IPV, but also sees the research gaps in theoretical explanations of IPHS (7). The most widely used model for understanding violence is the ecological model. It proposes a multifactorial explanation of causes including individual, relationship, community, and societal factors (7). Psychological explanations for IPHS have focused either on the homicide as the primary event with suicide as a subsequent response, or on suicide as the primary motive with the homicide making it easier to commit suicide (4, 8). Models of attachment theory and loss have also provided a psychological framework for understanding these violent acts in intimate relationships. The literature on IPV suggests a link between childhood attachment anxiety and insecure adult anxiety prompted by real or perceived separation from a partner (9, 10). These studies report that violent spouses, compared to nonviolent spouses, are more likely to experience abandonment anxiety in their relationships, be overly dependent on their partners, be jealous and untrusting, and have generally insecure attachment styles (11, 12).

A 2012 report on homicide-suicide in the Caribbean suggests that victim and offender profiles in Barbados, Guyana, Jamaica, and Trinidad were similar to those reported by the international literature (6). IPHS offenders are typically male, older than the victim, 44 years of age (median), married/cohabiting or recently separated, and many have no history of chronic domestic violence (1, 13). Epidemiological studies have identified triggers for IPHS that include jealousy, revenge over real or perceived infidelity, the breakdown of a relationship, a partner threatening separation, having a mental disorder, and financial and/or legal problems in the couple’s relationship (1, 2, 5, 14). Offenders can become obsessed with their spouse (characterized by stalking, control, and possessiveness), and if the partner desires to leave the relationship, the obsession becomes the source of passion, pain, and self-destruction (8).

Persons in intimate relationships who are characterized by mental health concerns, such as stress, violence, depression, and anxiety, often do not seek professional help, even for severe symptoms (15). This is particularly concerning since effective treatments for many mental health problems exist, even though accessibility to treatment may not be equitable (15). Incidence of mental health concerns in IPHS events is thought to be high, with few offenders or victims accessing mental health services during the relationship (5). Therefore, research on psychological and behavioral indicators of interpersonal conflict in homicide-suicide events can contribute to pertinent discussions on raising mental health awareness. Moreover, focusing on symptom recognition, at both the individual and community levels, will enhance public health strategies geared at prevention of domestic violence and death.

The objective of this study was to identify social and behavioral patterns and mental health concerns associated with IPHS in Jamaica through an analysis of media and police reports of IPHS incidents in 2007 – 2017.

## MATERIALS AND METHODS

An archival data review was conducted of police records and print, radio, and television reports of IPHS in Jamaica from January 2007 – June 2017. Given the challenges inherent in gathering data directly from individuals involved in homicide-suicide incidents, archival sources were a feasible alternative. Sources were police records and reports by major media houses. Media and police records often carry detailed, multiple accounts of homicide-suicide incidents given their infrequent occurrence and the general interest that they engender. To encompass several homicide-suicides, data were retrieved for 10 years. Only homicide-suicide events that involved persons who were in an intimate relationship at time of death, or previously had been, were included. Each homicide-suicide act counted as one piece of data, with all reports about a single incident collated and analyzed for relevant information.

For this study, an intimate relationship was defined as spousal partners in a heterosexual or homosexual relationship. IPHS was defined as an offender killing an intimate partner, with or without killing or causing fatal injury to other family members, and subsequently killing him/herself within 24 hours. A total of 27 such cases were reported during the study period; only one involved a homosexual relationship.

### Data extraction

A data retrieval sheet was used to record demographic information on the homicide offender/suicide victim and homicide victim, as well as relevant social and behavioral information. This data was based on eye-witness reports by family, community members, and/or police officers who had been at the crime scene, as reported by the media. Typically, news and police reports provide information from individuals who knew the deceased partners. Though verifying these accounts is not possible, other related studies have used similar methods to collect data (16).

Police reports were used to identify homicide-suicide incidents recorded during the study period and the dates on which these deaths occurred. The archives of media houses were then accessed and these dates were used to locate print, radio, and television reports. For each incident, the search started with the date on which the homicide-suicide occurred and continued until all subsequent media reports were exhausted. Each incident was assigned a unique identifier to protect the anonymity of the case. Multiple reports of the same incident were examined to verify the information collected earlier, as well as to provide new data. Information recorded in the data retrieval sheets for each incident was collated.

### Data analysis

The researchers created a list of descriptive codes ascribed to behaviors, relationships, and personality traits associated with homicide-suicide offenders and victims as described in the literature. These pre-identified codes were assigned to selected media extracts and used to organize the demographic and social data gathered. Open coding (labeling data based on the meaning that emerges) was applied to sections of the data not assigned a pre-identified code. Consensus on the codes and the data extracts to which they were assigned was reached by three researchers, two psychologists (AMP, NP) and one health educator (AB), after discussion of individually coded data. The data were fed into Dedoose^©^ analysis software (SocioCultural Research Consultants, LLC, Manhattan Beach, California, United States). A final list of codes and definitions was determined (Table 1) and used to identify emerging themes.

**FIGURE 1.**
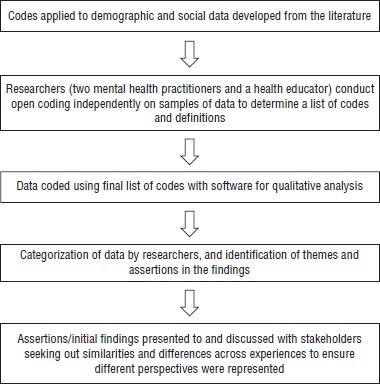
Flow chart of the process for analyzing data abstracts from media reports of intimate partner homicide-suicide in Jamaica, 2007 – 2017

***Source:*** Prepared by the authors.

To improve validity of the interpretations and findings, codes and themes were thoroughly discussed and agreed upon by all three investigators. These cross-validation and group interpretations were to reduce bias and increase the credibility and trustworthiness of the findings (17). Additionally, the findings were shared with stakeholders from the police force of Jamaica and media professionals. Their feedback was sought on any assertions and their implications. Figure 1 provides an overview of the data analysis process.

### Ethics

Ethics approval for the study was granted by the Ethics Committee of The University of the West Indies, Mona, Kingston, Jamaica (ECP 92 16/17). No identifying data were recorded from police or media records. Each homicide-suicide incident was given a unique numerical code to protect the anonymity of cases.

**TABLE 1.** Conceptualization of codes and their definitions for analyzing data on intimate partner homicide-suicide in Jamaica, 2007 – 2017

Code	Definition
Notable observations of behavior	Report of change in behavior or habits immediately prior to event
Relationship comments	
Positive	Descriptors indicating particularly happy, loving
Negative	Descriptors indicating abuse–verbal, physical etc.
Neutral	Descriptors indicating impersonal relating, no abuse, neither signs of particular closeness
Personality traits comments	
Positive	Descriptors of qualities that emphasize conflict resolution (i.e., peacemaker, quiet)
Negative	Descriptors of qualities that will aggravate relationships (i.e., quarrelsome, possessive)
Neutral	Descriptors of qualities that do not speak to intimate-relationship maintenance or aggravation (i.e., hard worker, loves his/her children)
Lifestyle diseases and mental illness	Descriptors of characteristics, i.e., substance use, history of mental illness

***Source:*** Prepared by the authors based on the study results.

## RESULTS

The archival data review of all reported homicide-suicide incidents between intimate partners in Jamaica over a 10-year period (2007–2017) identified 27 cases, resulting in a prevalence rate of 0.1 per 100 000. Males in a current relationship were the homicide-offender in all cases. Cases occurred in both rural and urban areas. Primarily guns (44%) and cutting instruments (44%) were the weapons in the homicides; guns (44%) and hanging (37%) were used in suicides. A pattern was identified for location and time of the incidents: most homicides and suicides occurred at home, from midnight – early morning or during the day. See Table 2 for a detailed description of the sample characteristics.

The findings that follow highlight salient characteristics as reported in our data review of male offenders, female victims, and the relationship experience of these intimate partners.

**TABLE 2.** Summary of descriptive characteristics of a sample of homicide-suicide events among intimate partners (*n* = 27), retrieved from police records and print, radio, and television reports by media houses in Jamaica, 2007 – 2017

Characteristics	Frequency of occurrence	Rate of occurrence (%)
Occupation (offender)		
Unskilled workers (farmer, mason)	10	37.1
Armed personnel (police, soldier)	9	33.3
Skilled workers (electrician, mechanic)	4	14.8
Professional (bank manager)	1	3.7
Business owner	1	3.7
Not reported	2	7.4

Occupation (victim)		
Unskilled worker (housekeeper, laborer)	3	11.1
Armed personnel (police, soldier)	3	11.1
Unemployed	3	11.1
Professional (bank manager, principal)	2	7.4
Business owner	2	7.4
Skilled worker (waitress)	1	3.7
Not reported	13	48.2

Location of homicide		
Home	16	59.3
Public space	7	25.9
Non-resident home	3	11.1
Not reported	1	3.7

Time occurred (homicide)		
Day time (6:05 a.m. – 6:00 p.m.)	10	37.1
Early morning (midnight – 6:00 a.m.)	9	33.3
Evening (6:05 p.m. – 11:55 p.m.)	4	14.8
Not reported	4	14.8

Location of suicide		
Home	15	55.6
Public space	8	29.6
Non-resident home	3	11.1
Not reported	1	3.7

Time occurred (suicide)		
Day time (6:05 a.m. – 6:00 p.m.)	10	37.1
Early morning (midnight – 6:00 a.m.)	9	33.3
Evening (6:05 p.m. – 11:55 p.m.)	4	14.8
Not reported	4	14.8

Weapon used (homicide)		
Instrument to cut/stab	12	44.4
Gun	12	44.4
Strangulation	2	7.4
Not reported	1	3.8

Weapon used (suicide)		
Gun	12	44.4
Strangulation (hanging)	10	37.1
Instrument to cut/stab	2	7.4
Fire	1	3.7
Drowning	1	3.7
Poisoning	1	3.7

Children orphaned		
Yes	16	59.3
No	3	11.1
Not reported	8	29.6

Knowledge of triggers for homicide		
Known triggers	23	85.2
Not known	0	0.0
Not reported	4	14.8

Cause of domestic disputes		
Not known	11	40.7
Jealousy/infidelity/obsession	6	22.2
Initiate separation	4	14.8
Money/house	2	7.5
Not reported	4	14.8

Circumstances prior to homicide		
Overt precipitant (saw spouse talking with a man, drinking alcohol, change in appearance, arguing)	10	37.1
Unexpected, element of surprise	6	22.2
Not reported	11	40.7

***Source:*** Prepared by the authors based on the study results.

### Characteristics of the male offender

The average offender tended to be 44 years (SD 10.47), older than his partner, and employed as armed security personnel (33%) or unskilled laborer (37%). Based on multiple print media reports, offenders were described by people in the community as having positive qualities: “always smiling,” “harmonious,” “affectionate,” “hard-working,” “well-mannered,” “never saw him angry,” and “personality would not cause alarm.” Any threat of harming the victim was seldomly documented. In almost one-quarter of the cases (22%), the homicide-suicide was described as unexpected. However, some community members had noted a “change in appearance” (in mood, behavior, or habit by the offender) immediately before the crime was committed.

Some family members were reported to describe offenders as abusive, jealous, controlling, possessive, and obsessive. One newspaper report stated “…[He] forbade the victim from talking to men…policed her use of social media…would search her phone and called her constantly.” Interviews with surviving family and friends, documented in both print and electronic media reports, portrayed some offenders as emotionally distraught: “appeared stressed and started crying during a conversation,” “had difficulty dealing with the separation,” and “had left a suicide note behind.”

### Characteristics of the victim

The average age of the victims was 34 years (SD 8.6). They were consistently described as quiet, and occasionally, as determined, caring, and having exceptional qualities such as, “exemplary educator and neighbor,” “outstanding individual,” and “talented, full of possibilities.” When media reports included the victim’s occupation, it varied: principal, executive, self-employed business woman, security officer, or unemployed.

Two victims had unsuccessfully reached out to the community and/or the police. Data from a radio interview indicated that one victim reached out to a neighbor who stated, “X called and told [me] they were having problems, but I didn’t expect this.” In another case, a newspaper report stated that the victim, a common law wife, tried to get assistance, “After the attack on the common law wife, she had alerted the police, but was told that she had to come into the station.”

### Characteristics of the intimate relationships

An age difference of 8 years or more was reported for almost one-half (44%) of the couples, with 26% documenting age differences of 15 – 27 years. In about one-quarter (26%) of the cases, ongoing conflict resulting in fighting was described. Behaviors depicting the male as obsessive about his partner and jealous were documented as the primary causes of the disputes. Police and electronic media reports documented family members’ descriptions of repeated attempts by female victims to end the relationship: “she wanted to end the relationship, but he wasn’t willing to give it up,” and “the wife sought a divorce, she reported being tired of the alleged abusive relationship.” Some neighbors’ reports were in contrast to reports of domestic disputes. In a print media account of an interview with a neighbor, a couple was described as “doing everything together, best friends, no history of fights, and not known to be troubled.” In more than one-half of the cases (59%), the male partner intentionally killed a mother, in some cases the mother of his child(ren), and then killed himself, thereby orphaning his children.

Of the 27 cases, there was only one report of a spouse seeking mental health counseling for the relationship. There were no records of community members, family, nor the police initiating help-seeking for the couples. Based on police reports, the female threatening to separate from her partner or initiating a separation was the immediate trigger for the homicide: “domestic dispute resulting from a breakdown in the couple’s relationship. The victim had decided to relocate to a nearby community.”

## DISCUSSION

To our knowledge, this is the first study to collect detailed information of IPHS patterns in a 10-year period in a Caribbean nation. Consistent with previous literature, we found the typical homicide-suicide offender to be a middle-aged male, older than his spouse, and directing violence at a female partner (1, 5). Female homicide victims were described as quiet, a trait that might have led them to remain in an unhealthy relationship until their death (16). Offenders were obsessive, jealous, and insecure (12, 16, 18). In contrast to the literature (7) on intimate partner homicide (IPH), reports of domestic violence between partners or threats to kill the spouse were not dominant. While IPHS and IPH share similar characteristics (14), our findings support some distinctions between the two.

The 2016 Global Homicide Report by the United Nations Office on Drugs and Crime ranked Jamaica as having the 6^th^ highest homicide rate in the world (19). Jamaica also struggles with violence against women by intimate partners (20, 21). Gun was the choice of weapon for both homicide and suicide in a sample of men who were employed primarily as armed security personnel or unskilled laborers. The frequent reports of gun use, particularly by armed security personnel, to murder a spouse/partner (1, 6) have prompted researchers to suggest policies that focus on effective firearm regulations to address homicide-suicide (4).

By all reports, the homicide-suicide events in this study were hidden acts that took the community by surprise. Most occurred in the privacy of a home and during hours when the act was least likely to be witnessed and/or prevented (2, 16, 22). The couples may have tried to conceal any trouble in their relationships from their neighbors, which would explain why neighbors’ comments regarding the homicide-offender were more favorable than comments by the family. This disparity in the comments may reflect “the charm of an abusive spouse” (23), who shows an affable side to his neighbors, while the family witnesses his controlling and jealous behaviors.

Although both partners may have attempted to mask conflicts in their relationship, the difficulties were noticed in the current sample. We found reports of men who were observed crying, appeared stressed and angry, displayed a sudden change in mood, and fought with their partners prior to the homicide. There were also reports of women who expressed a desire to leave the relationship, including a few who reached out to a neighbor or the police. Although these accounts were likely indicators of stress and depression (24), there was only one report of a spouse seeking professional mental health care. Additionally, community members did not seem to know what action to take in situations of domestic disputes.

In island states like Jamaica, although residents of communities are usually considered neighborly, spousal violence is regarded as domestic and private (25). Spousal violence has not resulted in a call-to-action by the wider community of family, friends, neighbors, and the police (26). Public campaigns that laud reporting domestic disputes to a police hotline as good neighborliness may help to combat a “right to privacy” response by neighbors. These campaigns could also raise awareness of available community resources, including legal, medical, counseling, and social services.

Community studies generally find a low rate of individuals seeking professional help for mental health (27). Even when health is affected by stress and strain, friends and relatives are the preferred source of help (28). While informal support has its benefits and ought to be encouraged, the advice of family members and religious leaders may sometimes deter a person from leaving an abusive relationship (29). The preference for getting help from friends and family is compounded by the stigma associated with mental illness (27, 30), a stigma that is pervasive among Caribbean people (31, 32).

In Small Island Developing States where mental health resources are scarce or not well utilized, preventative planning and efficacious use of resources should be a priority. Physicians, who are likely to be the first point of contact for spouses in domestic disputes, should be trained to detect signs of domestic violence, interpersonal conflict, and mental health concerns. They could use a simple three-item tool, such as the Partner Violence Screen (33), to screen for domestic violence symptoms. Other risk factors for homicide-suicide such as depression, suicidal behavior, substance/alcohol abuse, access to firearms, and a pending separation from a partner could also be probed (34). Implementing programs in schools to educate students on gender-based violence and building healthy relationships could further improve awareness. Also, members of the police force could benefit from clear institutional guidelines for responding to domestic disputes, and community liaising.

Changing how the media handles and reports incidents of IPHS should also be a focus. Rather than sensationalizing the incident, media reporters could alert the public to homicide-suicide patterns, dispel myths, and inform the community about responsible reporting.

Countries in the Caribbean should strengthen mental health services and increase access through clinics and community public settings. Strategies should be mobilized to change cultural attitudes toward gender relations and social norms that increase IPV risk. Le Franc suggests that in many Caribbean societies, domestic quarrels are viewed as “normal,” “deserved,” and a “challenge for women to overcome” because violence against women is entrenched in the culture (20). Furthermore, there are gender-based beliefs that allow for a man to be controlling and obsessively jealous of his partner (35); and other norms and stigma that inhibit men from seeking counseling.

IPHS is an issue necessitating specific attention for both women and men. Changing these socialization patterns, beliefs, and practices could align with the United Nation’s Sustainable Development Goals, specifically the targets that address gender inequality and mental health services and well-being. Future studies on cultural attitudes and responses to IPV need to encompass IPHS.

### Limitations

There is an inherent difficulty in retrieving data from documents that were not originally meant for research purposes. Specifically, obtaining information on social and mental health concerns and care-seeking behaviors was challenging as media and police reports do not routinely contain data on mental and social well-being. Case documentation may also have been impacted by inaccurate recall of events by persons providing information to the media or police. The sample size restricted additional quantitative analyses.

### Conclusions

Our findings concur with previously identified demographic and incident characteristics associated with IPHS. We have a broader understanding of the mental health concerns and the community response and responsibility surrounding these events in Jamaica. There are clear behavioral and mental health indicators that could prompt partners to seek help and encourage victims to seek protection.

Future research should examine the attitudes that family members, neighbors, and law enforcement officers have toward getting involved in spousal conflicts and what degree of influence they might have on help-seeking behavior. Educating the public on the characteristics and patterns that put individuals at risk for IPHS—the warning signs—should be integrated with existing community-based programs on violence and suicide prevention.

## Author contributions.

AMP conceived the original idea, analyzed the data, interpreted the results, wrote, and reviewed the paper. AB contributed analysis tools and analyzed the data, interpreted the results, wrote, and reviewed the paper. NP collected and analyzed the data, interpreted results, reviewed the paper. All authors reviewed and approved the final version.

## Acknowledgements.

The authors wish to acknowledge the Jamaica Constabulary Force Statistics and Information Management Unit, Ministry of National Security and Justice, Jamaica, for its assistance.

## Disclaimer.

Authors hold sole responsibility for the views expressed in the manuscript, which may not necessarily reflect the opinion or policy of the *RPSP/PAJPH* and/or PAHO.
